# Relapsing Fever Caused by *Borrelia lonestari* after Tick Bite in Alabama, USA

**DOI:** 10.3201/eid2902.221281

**Published:** 2023-02

**Authors:** Laia J. Vazquez Guillamet, Grace E. Marx, William Benjamin, Peter Pappas, Nicole A.P. Lieberman, Kimo Bachiashvili, Sixto Leal, Joshua A. Lieberman

**Affiliations:** University of Alabama, Birmingham, Alabama, USA (L.J. Vazquez Guillamet, W. Benjamin, P. Pappas, K. Bachiashvili, S. Leal);; Centers for Disease Control and Prevention, Fort Collins, Colorado, USA (G.E. Marx);; University of Washington School of Medicine, Seattle, Washington, USA (N.A.P. Lieberman, J.A. Lieberman)

**Keywords:** *Borrelia lonestari*, relapsing fever, tick, bacteria, parasites, vector-borne infections, zoonoses, Alabama, United States

## Abstract

We report an immunocompromised patient in Alabama, USA, 75 years of age, with relapsing fevers and pancytopenia who had spirochetemia after a tick bite. We identified *Borrelia lonestari* by using PCR, sequencing, and phylogenetic analysis. Increasing clinical availability of molecular diagnostics might identify *B. lonestari* as an emerging tickborne pathogen.

Tickborne diseases account for 77% of all vectorborne diseases reported in the United States, and incidence is increasing (*1*). The bacterium *Borrelia lonestari* was first detected in the lone star tick, *Amblyomma americanum,* in 1996 and has since been detected in both ticks and vertebrate hosts in many southeastern states (*2*,*3*). When first discovered, *B. lonestari* was a proposed etiology of southern tick-associated rash illness after *A. americanum* tick bites, but extensive efforts to isolate the bacterium in rash biopsies were unsuccessful (*4*). We report the detection of *B. lonestari* in a patient with febrile illness after a tick bite, demonstrating the potential of this bacterium as a human pathogen.

In late April 2019, a 75-year-old man in Alabama sought care at The Kirklin Clinic of the University of Alabama (Birmingham, AL, USA) for extreme fatigue and relapsing fevers accompanied by chills, sweating, headache, and dizziness that had recurred 1 or 2 times each week for the previous 4 weeks. He had a history of low grade follicular non-Hodgkin lymphoma and was receiving maintenance rituximab therapy. The patient had not traveled out of Alabama for several years but reported that he had removed an attached tick 4 weeks before symptom onset. The patient’s symptoms lasted ≈3 months before receiving a diagnosis. During this 3-month period, pancytopenia (lowest hemoglobin 9.3 g/dL; leukocytes, 2 × 10^9^ cells/L; platelets, 120 × 10^9^/L), mildly elevated alkaline phosphatase (216 IU/L), and mild hepatosplenomegaly, noted by computed tomography scan, developed in the patient. Physical examination revealed no skin lesions, lymphadenopathy, or organomegaly. Although the patient was evaluated by the oncology department for suspected recurrent lymphoma, the pathology laboratory reviewed a peripheral blood smear and reported the presence of spirochetes (Appendix Figure). Empirical oral treatment (100 mg doxycycline 2×/d) was initiated immediately. After the first dose, a fever developed in the patient, and he became stuporous. Emergent evaluation for an acute cerebrovascular stroke was negative. The patient returned to his baseline state of health within 24 hours and completed a full 10-day course of doxycycline without additional complications. His pancytopenia resolved in the subsequent months.

A blood sample from the patient was submitted to the University of Washington Medical Center Molecular Microbiology laboratory for broad-range bacterial PCR targeting the V1/V2 domains of the 16S rRNA gene (*5*). PCR yielded a high-quality 497-nt product (GenBank accession no. MN683828) that was sequenced and then classified by using BLAST (https://blast.ncbi.nlm.nih.gov) of sequences from GenBank and the reference laboratory’s sequence database. The PCR product had 100% identity to 3 published 16S rRNA gene sequences from *B. lonestari* (GenBank accession nos. AY166715, AY682921, and U23211) and 99.79% identity to another published 16S rRNA gene sequence from *B. lonestari* (GenBank accession no. AY682920) (Table). Percentage nucleotide identity of the patient sequence to other *Borrelia* spp. with high-confidence identifications was below the laboratory’s standard threshold of 99.7% for species-rank classification.

**Table:** Summary of BLAST results for 16S rRNA gene sequences used to clinically identify bacteria found in the patient’s blood sample in study of relapsing fever caused by *Borrelia lonestari* after tick bite in Alabama, USA*

Species	GenBank accession nos.	% Identity†	Confidence‡
*B. lonestari*	AY166715, AY682921, U23211	100%	Published
*B. lonestari*	AY682920	99.79%	Published
*B. turicatae*	NC_008710, NZ_CP015629	98.38%	RefSeq
*B. parkeri*	NZ_CP005851	98.38%	RefSeq
*B. venezuelensis*	MG651649	98.38%	Unpublished
*B. coriaceae*	NR_114544, NR_121718, NZ_CP005745	98.38%	Type
*B. miyamotoi*	AB900817, LC164096, LC164108	98.38%	Published
*B. hermsii*	NC_010673, NR_102957	97.78%	Type

*The V1/V2 hypervariable region of the 16S rRNA bacterial gene was amplified by PCR and BLAST (https://blast.ncbi.nlm.nih.gov) was used to align the patient’s sequences with reference sequences in GenBank. †Percentage identity with the 16S rRNA bacterial sequence from patient blood. ‡Confidence refers to level of curation for each reference sequence identified by using BLAST. Published indicates sequence record associated with peer-reviewed manuscript; RefSeq indicates records from curated GenBank RefSeq database (https://www.ncbi.nlm.nih.gov/refseq); Type indicates sequence from type material; unpublished indicates minimally curated direct submission to GenBank not associated with a peer-reviewed manuscript.

Because the clinical taxonomic classification was determined by a single locus, we retrospectively generated a single-locus phylogeny by using representative V1/V2 16S rRNA gene sequences to evaluate confidence in the clinical result (Figure) (*6*). The V1/V2 16S rRNA phylogenetic analysis appropriately distinguished between *Borrelia* spp. causing Lyme disease and relapsing fever. We observed 2 well-resolved groups, and the bacterial sequence from the patient sample formed a high-confidence clade with *B. lonestari* sequences (bootstrap >0.95) (Figure). The *B. lonestari* clade was well-separated from other homogenous clades of *Borrelia* spp. causing relapsing fever, which had similarly high-confidence bootstrap values. The 2 clades most closely related to *B. lonestari* were *B. miyamotoi* and *B. theilieri*, recapitulating published *Borrelia* relapsing fever group phylogeny (*7*).

**Figure:**
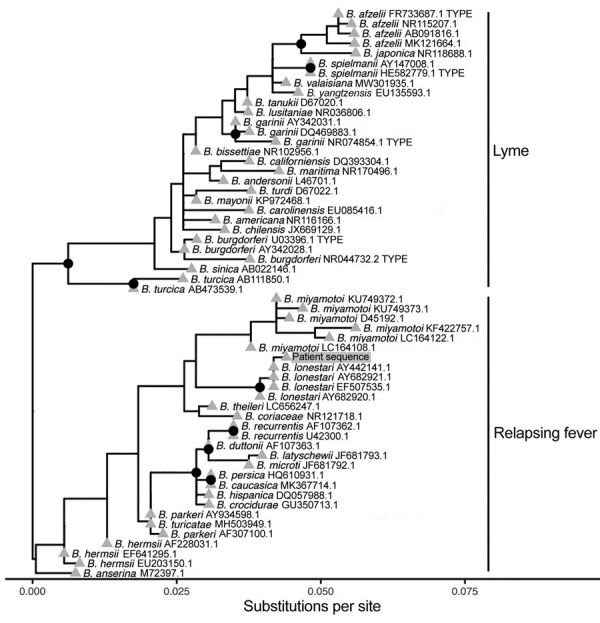
Phylogenetic analysis of bacterial sequence derived from patient’s blood (gray shading) in study of relapsing fever caused by *Borrelia lonestari* after tick bite in Alabama, USA. Phylogenetic tree was constructed from representative V1/V2 regions of 16S rRNA gene sequences from different *Borrelia* spp. known to cause Lyme disease or relapsing fever. GenBank accession numbers are indicated after the species names. The bacterial sequence from the patient sample formed a high-confidence clade with *B. lonestari* sequences and was most closely related to *B. miyamotoi*. Nodes with >95% confidence bootstrap values are labeled with black circles, and branch tips are labeled with gray triangles.

The first reported human case of *B. lonestari* infection was identified in an elderly patient with an erythema migrans-like rash that developed after an *A. americanum* tick bite; PCR identified *B. lonestari* in both a skin biopsy and the removed tick (*8*). In contrast, the patient we report had a longer illness and a pattern of relapsing fevers, absence of erythema migrans, and a possible Jarisch–Herxheimer reaction after initiation of antimicrobial therapy. *B. lonestari* has not been previously reported to cause tickborne relapsing fever, but this manifestation is not unexpected given the close homology to *B. miyamotoi*, the cause of hard tick relapsing fever (*9*). The patient’s immunocompromised condition from CD20 monoclonal antibody therapy might also have affected the patient’s clinical manifestations.

The infrequency of reported *B. lonestari* infections despite the frequent isolation of the bacteria from host-seeking *A. americanum* ticks suggests that this species might have a lower pathogenic potential than other *Borrelia* spp. more often associated with human disease. However, this assumption is hindered by the difficult isolation of *B. lonestari* through classic laboratory methods, including culturing (*2*,*10*). This case and associated sequence analysis highlight the clinical utility of molecular diagnostics for patients with suspected tickborne diseases. Increasing availability of molecular diagnostics might enable *B. lonestari* to be identified as an emerging tickborne pathogen, particularly causing opportunistic infections among persons who are immunocompromised.

Appendix Additional information for relapsing fever caused by *Borrelia lonestari* after tick bite in Alabama, USA.
