# Supplementary material for: Relapsing Fever Caused by Borrelia lonestari after Tick Bite in Alabama, USA
Source: Emerg Infect Dis. 2023 Feb;29(2):441–4. doi: 10.3201/eid2902.221281 (PMC9881794; doi:10.3201/eid2902.221281)
Supplement: Appendix — Additional information for relapsing fever caused by Borrelia lonestari after tick bite in Alabama, USA. [file 22-1281-Techapp-s1.pdf]

Article DOI: https://doi.org/10.3201/eid2902.221281

Relapsing Fever Caused by *Borrelia lonestari* after Tick Bite in Alabama, USA

Appendix

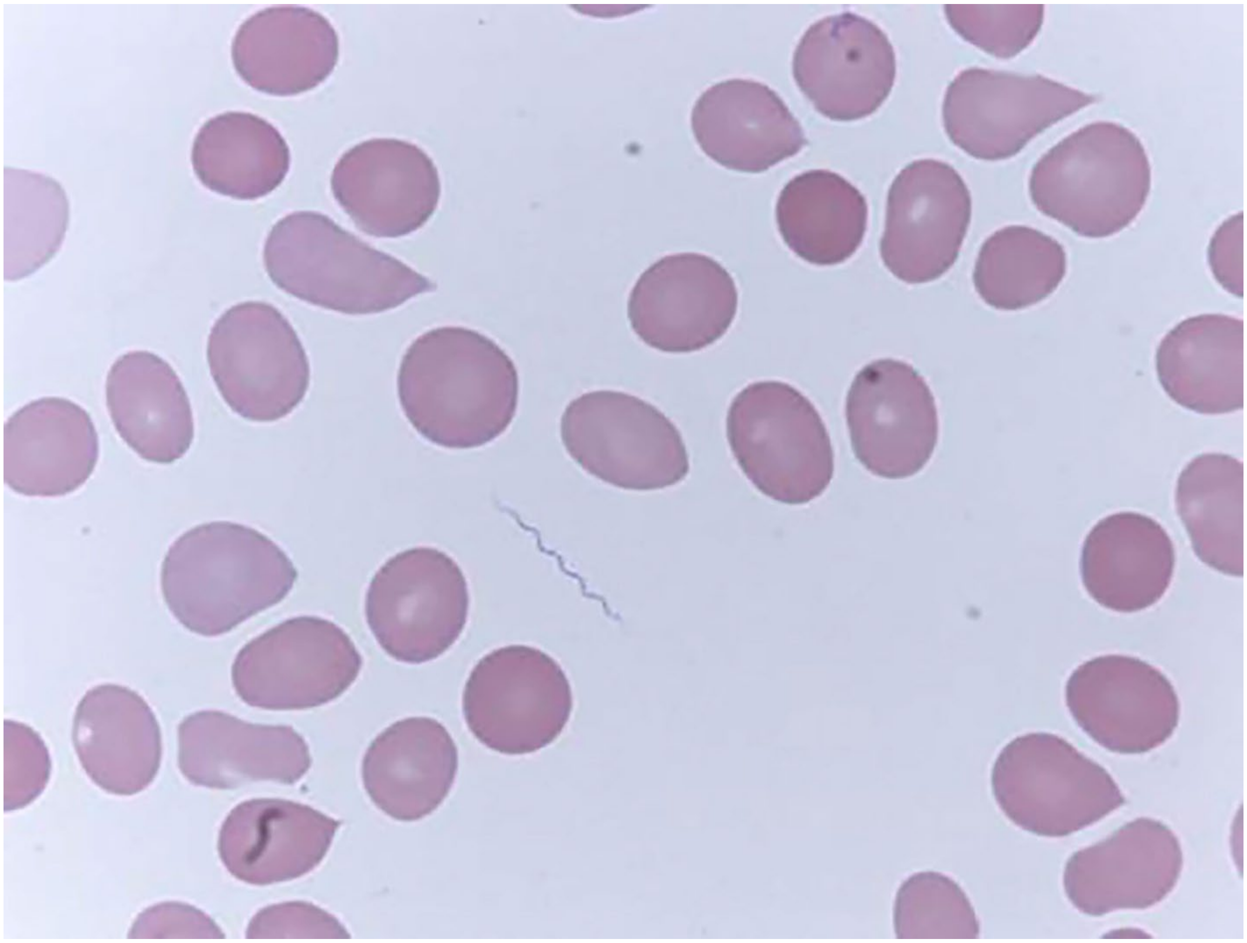

Appendix Figure. Microscopic image of spirochete bacteria found in patient's peripheral blood in study of relapsing fever caused by *Borrelia lonestari* after tick bite in Alabama, USA. Peripheral blood was collected before treatment with the antimicrobial drug doxycycline and smears were prepared for microscopic examination, original magnification ×1,000.
